# Supplementary material for: A Likelihood Approach for Real-Time Calibration of Stochastic Compartmental Epidemic Models
Source: PLoS Comput Biol. 2017 Jan 17;13(1):e1005257. doi: 10.1371/journal.pcbi.1005257 (PMC5240920; doi:10.1371/journal.pcbi.1005257)
Supplement: S1 File — (TAR.GZ) [file pcbi.1005257.s014.tar.gz › HSPH_Online-SI-Revision/PF08/n1-severe/n1-severe_PF_table0.pdf]

1	R0	{1.51081, 13.7078}	{1.65075, 14.0215}	{{1.24636, 7.46722}, {2.20441, 19.6265}}	{20.9617, 26.6006}	inf	54.	65.4096	{15.1913, 126.293}	63.0987	0.265733	newly	34.	29.62	{10., 52.}	42.1176	0.245478	Reff	1.05303	0.845415	{0.500603, 1.15779}	25.0321	ar	656.	754.12	{526., 985.}	24.3018
2	R0	{1.73182, 11.3873}	{1.69245, 14.3013}	{{1.24852, 7.01378}, {2.30341, 20.}}	{21.2933, 42.5955}	inf	80.	87.6239	{25.6057, 159.706}	54.6986	0.256136	newly	53.	39.98	{18., 69.}	38.1509	0.227402	Reff	1.15166	0.89048	{0.528393, 1.18706}	25.5292	ar	653.	772.52	{512., 992.}	26.4717
3	R0	{1.50449, 10.3474}	{1.69501, 14.4729}	{{1.191, 8.13391}, {2.45872, 20.}}	{24.8261, 51.2764}	inf	60.	77.9681	{25.3882, 141.945}	68.2307	0.255779	newly	43.	35.54	{14., 59.}	44.3256	0.244538	Reff	1.08474	0.901668	{0.512609, 1.2107}	23.7976	ar	651.	758.5	{492., 1018.}	28.3287
4	R0	{1.63493, 9.1955}	{1.73249, 13.468}	{{1.18979, 6.05643}, {2.31231, 18.9003}}	{23.1542, 58.3073}	inf	87.	77.2328	{5.27011, 142.666}	58.495	0.433213	newly	59.	35.7	{4., 73.}	49.7966	0.486991	Reff	1.05126	0.921235	{0.555024, 1.27995}	27.3314	ar	663.	626.53	{306., 876.}	26.7195
5	R0	{1.53822, 8.05553}	{1.70873, 13.2802}	{{1.14345, 6.63253}, {2.31231, 18.9248}}	{24.7777, 73.276}	inf	55.	69.8481	{4.08797, 154.409}	86.1078	0.443205	newly	40.	31.05	{3., 64.}	54.375	0.421271	Reff	1.01061	0.855429	{0.514134, 1.17897}	26.3681	ar	621.	609.5	{249., 867.}	30.5282
6	R0	{1.69374, 17.0793}	{1.63076, 14.8031}	{{1.19751, 7.13703}, {2.24144, 20.}}	{20.0618, 23.42}	inf	97.	73.7177	{17.445, 162.943}	53.0661	0.363138	newly	40.	30.62	{12., 57.}	44.95	0.288132	Reff	1.25676	0.847751	{0.546616, 1.14479}	32.7159	ar	662.	735.98	{487., 968.}	24.2538
7	R0	{1.42179, 11.8031}	{1.66833, 14.6928}	{{1.19278, 8.08825}, {2.34585, 19.955}}	{25.0069, 37.5856}	inf	57.	72.9219	{23.4174, 124.904}	63.5089	0.263999	newly	30.	31.12	{12., 53.}	43.6	0.208317	Reff	0.963974	0.783251	{0.436999, 1.06508}	23.951	ar	517.	763.15	{509., 1000.}	49.6228
8	R0	{1.46683, 6.95875}	{1.88325, 13.7889}	{{1.25721, 6.88118}, {2.59602, 19.1171}}	{34.4726, 102.363}	inf	92.	90.3725	{12.422, 150.117}	48.9054	0.322584	newly	97.	41.57	{9., 73.}	57.268	0.499123	Reff	0.815557	0.851281	{0.440142, 1.26723}	31.1236	ar	653.	695.36	{402., 899.}	24.1807
9	R0	{1.34525, 11.505}	{1.71742, 14.6454}	{{1.2005, 7.85158}, {2.3112, 20.}}	{32.1324, 40.8388}	inf	47.	74.202	{17.5966, 148.697}	86.955	0.297944	newly	31.	31.29	{12., 57.}	44.2258	0.232876	Reff	1.01701	0.779645	{0.442444, 1.07349}	27.3238	ar	532.	779.86	{517., 1004.}	49.1429
10	R0	{1.77958, 14.372}	{1.68183, 15.5856}	{{1.21101, 10.4783}, {2.5548, 20.}}	{23.6991, 24.5374}	inf	110.	78.7194	{31.1324, 149.221}	47.4508	0.295132	newly	47.	31.99	{12., 56.}	43.5957	0.269417	Reff	0.948516	0.802499	{0.420293, 1.08287}	22.7147	ar	671.	761.27	{512., 1013.}	25.2235
11	R0	{1.60334, 19.9548}	{1.6204, 13.908}	{{1.20583, 7.2329}, {2.22633, 19.9329}}	{19.6055, 30.3452}	inf	94.	43.2681	{4.33019, 92.2328}	58.768	0.553754	newly	31.	18.7	{4., 35.}	50.	0.401728	Reff	1.08867	0.703425	{0.4029, 0.930998}	35.6973	ar	617.	730.79	{483., 966.}	28.1896
12	R0	{1.68623, 7.58585}	{1.89, 13.8882}	{{1.27627, 8.42022}, {2.73737, 19.255}}	{25.2456, 87.7116}	inf	74.	95.8837	{22.9309, 183.351}	70.8603	0.30338	newly	59.	44.76	{12., 75.}	39.2542	0.276245	Reff	0.881898	0.854452	{0.511298, 1.22807}	25.9037	ar	640.	693.42	{355., 907.}	26.8906
13	R0	{1.82689, 18.0022}	{1.69259, 14.6668}	{{1.25701, 7.40524}, {2.34792, 20.}}	{20.7255, 23.8115}	inf	136.	78.6105	{24.1104, 170.861}	53.0606	0.413499	newly	49.	33.05	{12., 64.}	45.8163	0.323929	Reff	1.10344	0.818013	{0.502464, 1.14443}	28.1888	ar	673.	769.78	{520., 1004.}	23.1857
14	R0	{1.75911, 9.16883}	{1.7793, 13.2064}	{{1.14345, 6.31117}, {2.59755, 19.1171}}	{25.9881, 56.6326}	inf	93.	80.3269	{5.85859, 165.059}	53.8999	0.42349	newly	75.	37.9	{4., 67.}	51.76	0.51594	Reff	1.16629	0.952164	{0.550298, 1.3489}	30.7109	ar	661.	631.1	{238., 907.}	30.1089
15	R0	{1.46168, 11.3859}	{1.73358, 14.5809}	{{1.27046, 8.29879}, {2.34411, 19.984}}	{24.4358, 39.9403}	inf	70.	69.1884	{13.3733, 137.367}	53.8926	0.311363	newly	36.	29.23	{8., 56.}	44.9167	0.283755	Reff	0.809771	0.758617	{0.447586, 1.04193}	23.625	ar	556.	791.06	{572., 1018.}	44.0396
16	R0	{1.53577, 11.7581}	{1.72211, 13.5307}	{{1.13239, 6.25107}, {2.31344, 19.0531}}	{25.7609, 35.1067}	inf	94.	77.3085	{7.29603, 161.354}	55.2372	0.423233	newly	55.	36.52	{5., 66.}	46.1818	0.419266	Reff	1.05507	1.01864	{0.63546, 1.54534}	27.0833	ar	625.	622.72	{251., 878.}	30.5728
17	R0	{1.70698, 9.72924}	{1.85316, 13.1728}	{{1.13176, 6.8071}, {2.5458, 18.2464}}	{25.8587, 47.8783}	inf	101.	93.2274	{8.85979, 181.402}	50.9528	0.356404	newly	63.	43.75	{6., 75.}	41.9524	0.335538	Reff	0.913234	0.867159	{0.42669, 1.30629}	27.0095	ar	675.	670.85	{242., 892.}	27.5748
18	R0	{1.5973, 14.931}	{1.63549, 12.8964}	{{1.13176, 5.71232}, {2.22212, 19.0443}}	{21.7401, 29.6089}	inf	53.	66.6391	{2.36984, 144.339}	91.107	0.508677	newly	23.	32.48	{3., 65.}	92.6957	0.448515	Reff	1.28583	1.00232	{0.653594, 1.41937}	30.2444	ar	632.	584.9	{248., 841.}	28.4589
19	R0	{1.87468, 17.004}	{1.67743, 14.8222}	{{1.19754, 7.37673}, {2.38759, 20.}}	{24.5878, 22.585}	inf	112.	59.1215	{12.7707, 130.037}	55.2153	0.433345	newly	45.	23.57	{8., 41.}	50.7778	0.368977	Reff	0.987956	0.703632	{0.397702, 0.985078}	29.7182	ar	692.	758.04	{496., 1021.}	23.7197
20	R0	{1.84715, 12.531}	{1.67426, 14.7505}	{{1.19363, 7.39989}, {2.2469, 20.}}	{21.4317, 34.9745}	inf	90.	63.0762	{10.6545, 135.332}	54.3269	0.401862	newly	55.	25.46	{8., 47.}	57.0545	0.455217	Reff	0.936505	0.73259	{0.438653, 1.00003}	25.7042	ar	661.	750.88	{482., 980.}	25.1891
21	R0	{1.75938, 13.5959}	{1.70821, 13.6751}	{{1.13239, 5.72868}, {2.42687, 19.0531}}	{22.5553, 28.0372}	inf	92.	78.6318	{4.61248, 161.354}	57.3344	0.459759	newly	50.	36.2	{4., 74.}	48.56	0.435319	Reff	1.1911	0.975406	{0.63546, 1.5291}	29.0681	ar	620.	618.19	{255., 882.}	30.1887
22	R0	{1.63456, 10.7469}	{1.75513, 15.0457}	{{1.22837, 7.60642}, {2.37573, 20.}}	{20.8491, 50.4312}	inf	85.	84.0883	{17.8227, 163.2}	55.8437	0.314268	newly	61.	33.23	{10., 61.}	49.0656	0.370935	Reff	0.81728	0.74432	{0.457273, 1.01381}	22.4408	ar	654.	796.04	{570., 996.}	28.2171
23	R0	{1.62702, 19.1392}	{1.64108, 12.5625}	{{1.11461, 5.71232}, {2.43143, 18.9813}}	{25.0436, 34.7936}	inf	97.	42.669	{2.25622, 95.6764}	60.7363	0.671745	newly	37.	19.33	{2., 41.}	56.2432	0.538732	Reff	1.02014	0.780093	{0.364998, 1.0551}	31.9669	ar	600.	574.85	{259., 877.}	32.6217
24	R0	{1.70466, 12.1202}	{1.81316, 14.5464}	{{1.29694, 8.26022}, {2.35193, 20.}}	{20.8232, 33.7126}	inf	70.	43.6277	{15.1365, 88.8306}	52.5159	0.356469	newly	32.	18.86	{8., 35.}	48.75	0.323684	Reff	0.946086	0.86171	{0.559536, 1.21811}	23.8662	ar	613.	788.37	{541., 977.}	34.6248
25	R0	{1.61735, 11.3169}	{1.65776, 15.1772}	{{1.25521, 8.42265}, {2.24203, 20.}}	{17.8636, 44.0265}	inf	103.	59.3611	{9.89802, 117.07}	50.6769	0.427331	newly	62.	24.68	{5., 42.}	60.4839	0.544972	Reff	0.826466	0.716323	{0.461217, 0.963639}	22.3486	ar	681.	754.44	{533., 963.}	21.3216
26	R0	{1.56977, 12.3035}	{1.69802, 13.0878}	{{1.12977, 5.32185}, {2.43839, 18.9248}}	{25.16, 33.6382}	inf	78.	66.2453	{2.53207, 142.61}	65.4183	0.53838	newly	42.	29.3	{2., 64.}	55.0952	0.511695	Reff	1.03605	0.853354	{0.511557, 1.17085}	26.9637	ar	594.	594.9	{240., 879.}	33.8418
27	R0	{1.46317, 5.8298}	{1.73621, 13.2611}	{{1.15964, 6.62607}, {2.31231, 19.0269}}	{27.9049, 131.889}	inf	64.	68.2724	{3.34324, 163.864}	78.9366	0.490099	newly	64.	31.25	{3., 66.}	58.6719	0.59123	Reff	0.847175	0.820994	{0.447029, 1.13664}	26.053	ar	586.	636.4	{300., 888.}	31.0614
28	R0	{1.70002, 14.9806}	{1.75348, 13.5538}	{{1.12977, 6.05643}, {2.42687, 19.2972}}	{24.1853, 25.9932}	inf	110.	80.9429	{7.29603, 165.059}	54.5816	0.457049	newly	42.	37.52	{8., 74.}	50.7143	0.377568	Reff	1.16281	0.984615	{0.575745, 1.5291}	30.4925	ar	652.	630.34	{269., 878.}	29.2117
29	R0	{1.52729, 16.1014}	{1.7021, 14.797}	{{1.21749, 8.04186}, {2.40161, 20.}}	{24.6552, 21.7986}	inf	86.	55.7931	{11.6327, 112.302}	49.5706	0.361333	newly	44.	23.09	{7., 43.}	50.4318	0.394886	Reff	0.925538	0.705376	{0.344666, 1.03093}	28.4032	ar	592.	762.61	{514., 1022.}	34.0591
30	R0	{1.72795, 13.5635}	{1.70139, 14.2188}	{{1.21458, 8.01351}, {2.36663, 20.}}	{20.966, 27.2628}	inf	92.	67.8205	{9.37342, 138.305}	52.2603	0.382784	newly	43.	28.83	{8., 55.}	49.4186	0.360052	Reff	0.865703	0.798054	{0.44312, 1.17995}	26.4212	ar	668.	771.61	{524., 974.}	26.2799
31	R0	{1.51591, 18.1544}	{1.70909, 14.1727}	{{1.21724, 6.9864}, {2.38845, 19.9732}}	{24.1766, 25.6155}	inf	59.	65.8042	{9.23183, 127.246}	66.3016	0.330095	newly	16.	27.47	{7., 54.}	96.9375	0.296883	Reff	1.1051	0.802854	{0.474458, 1.10718}	28.9089	ar	541.	766.99	{509., 987.}	44.7043
32	R0	{1.4282, 10.3534}	{1.73186, 14.3601}	{{1.207, 7.40144}, {2.41171, 20.}}	{28.6658, 50.9397}	inf	38.	89.4243	{26.6081, 231.183}	149.032	0.345393	newly	21.	39.01	{15., 80.}	99.1905	0.273689	Reff	1.1397	0.884428	{0.498493, 1.17988}	26.0083	ar	521.	774.43	{501., 1024.}	50.8196
33	R0	{1.56693, 11.3972}	{1.75344, 13.4413}	{{1.08887, 7.10524}, {2.46533, 18.6082}}	{29.4036, 34.4195}	inf	107.	84.8595	{8.33989, 177.453}	51.5299	0.396572	newly	62.	40.46	{6., 75.}	45.7419	0.380027	Reff	0.965229	0.929498	{0.553148, 1.2885}	26.3214	ar	695.	628.58	{254., 884.}	28.5353
34	R0	{1.50675, 19.8823}	{1.7065, 13.6911}	{{1.22127, 6.40844}, {2.38326, 20.}}	{26.024, 31.2841}	inf	68.	41.1084	{8.71182, 71.9931}	52.996	0.40523	newly	22.	18.02	{5., 33.}	44.8182	0.27808	Reff	0.90405	0.725462	{0.422764, 1.02937}	26.6067	ar	587.	750.17	{466., 996.}	36.3424
35	R0	{1.72889, 7.76068}	{1.85938, 13.9162}	{{1.24751, 7.55154}, {2.46533, 18.6082}}	{23.7606, 84.6899}	inf	77.	97.7469	{15.0364, 179.011}	70.0778	0.353567	newly	62.	45.18	{10., 78.}	39.871	0.311924	Reff	1.04771	0.888509	{0.551954, 1.3489}	28.9746	ar	650.	685.33	{327., 885.}	26.9308
36	R0	{1.62644, 4.12303}	{1.92645, 12.8897}	{{1.21725, 7.10524}, {2.59602, 18.2127}}	{30.7309, 212.73}	inf	43.	103.037	{15.8867, 200.204}	161.913	0.42866	newly	57.	49.03	{12., 83.}	42.4035	0.276934	Reff	0.769306	0.805651	{0.494077, 1.12941}	28.0316	ar	652.	698.81	{333., 906.}	29.0598
37	R0	{1.70878, 15.8072}	{1.66521, 14.6986}	{{1.22699, 7.77119}, {2.18036, 20.}}	{18.8874, 22.9083}	inf	84.	45.2092	{11.6364, 92.8351}	53.9588	0.447338	newly	36.	19.05	{5., 35.}	52.5833	0.420862	Reff	0.915906	0.672502	{0.37528, 0.888741}	28.2341	ar	674.	761.19	{528., 970.}	23.2122
38	R0	{1.58967, 6.80568}	{1.86716, 13.6535}	{{1.13227, 7.10524}, {2.47514, 19.1171}}	{30.3865, 105.421}	inf	73.	95.0493	{9.10989, 175.311}	75.7095	0.377903	newly	63.	43.05	{6., 79.}	43.6667	0.357372	Reff	0.86637	0.802856	{0.475129, 1.16844}	26.1656	ar	610.	682.92	{287., 891.}	33.0361
39	R0	{1.344, 8.19498}	{1.70372, 14.988}	{{1.20807, 7.36979}, {2.37526, 20.}}	{30.8623, 89.3308}	inf	39.	72.9974	{17.8068, 145.241}	108.104	0.321618	newly	36.	29.67	{11., 52.}	40.1944	0.250215	Reff	0.928704	0.77312	{0.438981, 1.06684}	24.5019	ar	551.	766.48	{504., 1003.}	42.2178
40	R0	{1.61509, 7.90825}	{1.8035, 12.6284}	{{1.11228, 6.55128}, {2.51417, 17.5607}}	{28.408, 68.8179}	inf	48.	85.8958	{4.63863, 170.722}	122.83	0.468954	newly	41.	41.99	{5., 81.}	57.3902	0.357158	Reff	1.04173	0.914289	{0.461493, 1.32039}	29.1218	ar	581.	646.02	{222., 906.}	38.0103
41	R0	{1.60017, 5.90605}	{1.88604, 13.9524}	{{1.22252, 8.23415}, {2.52651, 19.0531}}	{27.4996, 137.157}	inf	76.	92.7826	{23.81, 184.451}	65.0836	0.303617	newly	76.	42.48	{15., 75.}	50.0789	0.371631	Reff	0.832088	0.83919	{0.430334, 1.22408}	29.4578	ar	661.	708.65	{443., 891.}	22.7035
42	R0	{1.64436, 15.1337}	{1.63356, 12.8429}	{{1.14096, 6.18069}, {2.22832, 18.9813}}	{22.8681, 29.337}	inf	74.	60.2871	{3.07111, 124.378}	67.3608	0.526691	newly	39.	26.97	{2., 61.}	57.2564	0.468852	Reff	1.11323	0.901114	{0.582897, 1.17085}	28.7282	ar	640.	578.85	{240., 850.}	30.3453
43	R0	{1.48917, 11.917}	{1.57992, 13.7474}	{{1.18762, 6.68459}, {2.0959, 19.9911}}	{18.9767, 36.9414}	inf	72.	41.3419	{4.59339, 83.7955}	53.0908	0.486968	newly	36.	18.05	{4., 32.}	52.6389	0.458803	Reff	0.927753	0.707759	{0.432257, 0.934555}	25.4028	ar	551.	709.88	{499., 955.}	33.6806
44	R0	{1.65577, 7.89317}	{1.75295, 13.6666}	{{1.18939, 8.04006}, {2.31174, 18.9248}}	{23.3681, 79.4035}	inf	65.	76.1725	{5.27011, 142.666}	73.0164	0.416346	newly	60.	34.96	{4., 68.}	50.3333	0.49596	Reff	1.09446	0.924577	{0.552252, 1.45389}	30.0687	ar	576.	635.69	{306., 873.}	36.0156
45	R0	{1.6909, 12.3275}	{1.68481, 13.1957}	{{1.14345, 6.78013}, {2.22832, 18.9248}}	{21.9185, 31.3773}	inf	82.	55.8098	{4.29022, 127.359}	60.2643	0.515539	newly	47.	24.69	{4., 49.}	57.8085	0.508911	Reff	0.968886	0.766755	{0.448988, 1.02832}	28.6148	ar	675.	603.05	{274., 857.}	27.2104
46	R0	{1.62351, 16.0698}	{1.63382, 13.9707}	{{1.24492, 8.18447}, {2.30444, 20.}}	{20.3688, 25.4254}	inf	58.	61.484	{15.7671, 104.492}	55.5825	0.270659	newly	28.	27.58	{12., 51.}	46.	0.231328	Reff	1.25173	0.798496	{0.444406, 1.1006}	36.2282	ar	591.	731.91	{487., 963.}	31.8291
47	R0	{1.52372, 4.38599}	{1.89153, 13.247}	{{1.32613, 8.33653}, {2.68687, 18.765}}	{31.893, 203.168}	inf	71.	97.7018	{13.7882, 203.774}	82.7237	0.353946	newly	95.	48.56	{8., 91.}	50.7579	0.429378	Reff	0.888329	0.971601	{0.573538, 1.39412}	30.7661	ar	618.	693.7	{434., 909.}	28.4919
48	R0	{1.44306, 11.788}	{1.59719, 12.2765}	{{1.12882, 4.64686}, {2.20345, 18.9086}}	{24.8026, 36.3258}	inf	70.	38.9212	{1.12514, 89.2441}	64.456	0.70159	newly	36.	16.63	{1., 34.}	60.3611	0.642645	Reff	0.883153	0.743098	{0.42064, 1.03942}	26.2311	ar	534.	569.45	{260., 860.}	36.0805
49	R0	{1.63624, 11.3893}	{1.80769, 13.457}	{{1.21278, 6.62607}, {2.59602, 19.0269}}	{26.8481, 36.7267}	inf	91.	83.1226	{8.37775, 150.117}	53.0591	0.348732	newly	55.	37.75	{9., 72.}	45.4364	0.339256	Reff	0.927748	0.833086	{0.466299, 1.22832}	27.6875	ar	643.	661.87	{370., 896.}	26.6983
50	R0	{1.46113, 11.1322}	{1.72381, 14.8174}	{{1.20531, 7.77351}, {2.43183, 20.}}	{27.4228, 44.7133}	inf	63.	90.211	{28.0007, 195.08}	76.8787	0.262507	newly	52.	38.75	{16., 72.}	47.3654	0.27957	Reff	1.09731	0.903477	{0.544317, 1.18662}	23.5202	ar	501.	765.32	{450., 1010.}	56.3313
